# A Narrative Review of Psychological and Physical Rehabilitation in Infertility Treatment: Toward Holistic Outcomes Beyond Conception

**DOI:** 10.3390/medicina62071335

**Published:** 2026-07-11

**Authors:** Muhammad Adib Dwi Tamma Putra, Muhammad Ayub Endratamma, Muhammad Fadill Akbar, Adila A. Hamid, Mohd Helmy Mokhtar

**Affiliations:** 1 Department of Physical Medicine and Rehabilitation, Faculty of Medicine, University of Sriwijaya, Palembang 30128, Indonesia; adibdwi@unsri.ac.id; 2 Doctoral Program, AMA University, Quezon City 1106, Philippines; 3 Faculty of Medicine, Universitas Sriwijaya, Palembang 30128, Indonesia; muhamadayubendratama@gmail.com (M.A.E.); mfadillakbar@unsri.ac.id (M.F.A.); 4 Department of Physiology, Faculty of Medicine, Universiti Kebangsaan Malaysia, Cheras, Kuala Lumpur 56000, Malaysia; adilahamid@ukm.edu.my

**Keywords:** infertility, rehabilitation, cognitive behavioral therapy, exercise therapy, quality of life

## Abstract

Infertility is a complex health issue that extends beyond biological mechanisms, encompassing significant psychological, relational, and social aspects. Advances in assisted reproductive technologies have improved the chances of conception, yet many couples discontinue treatment due to stress, stigma, and reduced quality of life. This narrative review explores the role of rehabilitation as a complementary approach to conventional fertility care, highlighting psychological, physical, and integrated interventions. Psychological rehabilitation strategies, such as cognitive behavioral therapy, mindfulness-based interventions, and couple-focused counseling, have been shown to reduce anxiety and depression, improve coping, and, in some cases, support better adherence to fertility treatments. Physical rehabilitation approaches, including pelvic floor muscle training, structured exercise for women with polycystic ovary syndrome, lifestyle and fitness interventions for men, yoga, and sexual rehabilitation, contribute to reproductive health, reduce pain, and enhance intimacy. Integrated models that combine psychological and physical components show the greatest promise, as they address the interaction between stress, physical function, and reproductive outcomes. Overall, the evidence suggests that infertility care should not focus solely on achieving conception. Rehabilitation offers opportunities to strengthen emotional resilience, improve sexual health, and support relationship stability, ensuring that patients benefit not only medically but also in terms of long-term well-being and quality of life.

## 1. Introduction

Infertility is recognized as both a medical condition and a complex social and emotional experience. Clinically, infertility is defined as the inability to achieve pregnancy after twelve months of regular, unprotected intercourse, or after six months for women over age 35, reflecting the age-related decline in ovarian reserve and reproductive capacity [1,2]. Epidemiologically, infertility is a significant global health concern, affecting approximately 10–15% of couples of reproductive age worldwide [3]. However, its prevalence varies by geographic location, socioeconomic status, and access to healthcare [4]. This prevalence highlights infertility as both a clinical issue and a major public health concern, affecting population dynamics, healthcare resource allocation, and societal welfare.

Traditional management has primarily focused on identifying and correcting underlying physiological abnormalities. Key biomedical targets including ovulatory dysfunction, tubal obstruction, endometriosis, uterine abnormalities, and male factor conditions, remain the main areas for investigation and treatment [5]. In recent decades, the development of assisted reproductive technologies (ART), such as in vitro fertilization (IVF) and intracytoplasmic sperm injection (ICSI), has transformed fertility care [6]. Despite these advancements, infertility cannot be fully understood or managed through biology alone. Infertility involves not only physiological issues but also significant psychological and social aspects. For many individuals and couples, the inability to conceive profoundly affects identity, intimacy, and social belonging [7,8]. These broader impacts underscore the need for more comprehensive, patient-centered models of care that address both the physical and psychosocial consequences of infertility, highlighting the limitations of a solely biomedical approach.

From a rehabilitation perspective, infertility is understood not only as the inability to achieve pregnancy but also as an impairment of reproductive function. According to the World Health Organization’s International Classification of Functioning, Disability and Health (ICF), health conditions should be evaluated not only by diagnosis but also by their effects on body functions and structures, activities, participation, and contextual factors [9]. Within this framework, infertility affects body functions through disturbances in reproductive and sexual function, including ovulatory dysfunction, male-factor infertility, hormonal abnormalities, and infertility-associated sexual dysfunction [2,10,11,12]. These impairments may contribute to activity limitations in sexual intimacy, fertility-related decision making, treatment adherence, and stress management, as well as participation restrictions affecting marital relationships, family roles, social interactions, and community engagement [5,10,13,14]. In addition, environmental factors such as cultural expectations regarding parenthood, social stigma, healthcare accessibility, and financial burden may further influence functioning and participation [10,14,15], while personal factors including resilience, coping style, reproductive identity, self-efficacy, and psychological well-being affect individual adaptation to infertility [16,17,18,19]. Therefore, infertility should be understood not only as a reproductive disorder but also as a condition with multidimensional consequences for functioning and participation.

This broader conceptualization provides the rationale for applying rehabilitation principles to infertility care. Contemporary rehabilitation is defined as a set of interventions designed to optimize functioning and reduce disability in individuals with health conditions in interaction with their environment [20]. As emphasized in major Physical and Rehabilitation Medicine frameworks, rehabilitation extends beyond restoring lost function to include maintaining function, preventing secondary complications, promoting participation, enhancing autonomy, and improving quality of life throughout the continuum of care [21]. Therefore, rehabilitation in infertility should not be viewed solely as an adjunct strategy to improve pregnancy rates. Instead, it represents a biopsychosocial approach aimed at optimizing reproductive and sexual functioning, supporting psychological well-being, strengthening interpersonal relationships, facilitating social participation, and improving overall quality of life. This perspective provides the theoretical foundation for integrating psychological, physical, sexual, and lifestyle rehabilitation interventions into comprehensive infertility management.

A substantial body of evidence shows that people undergoing fertility assessment or treatment experience significantly higher levels of psychological distress compared to their fertile counterparts [22]. Depression and anxiety are particularly common and are often worsened by repeated treatment cycles, uncertainty about outcomes, social stigma, or strained relationships [23,24,25]. Men and women may experience and express this distress differently, but both are vulnerable to its effects. Gender differences are evident in both the experience and expression of distress; women often report higher levels of emotional discomfort, while men may be less likely to disclose psychological challenges even when experiencing similar internal stress [15,26]. Importantly, individual mental health is not the only aspect affected; infertility also disrupts couple dynamics, intimacy, and, depending on cultural context, community relationships and social standing [27,28].

Despite advances in ART, treatment discontinuation remains a significant and persistent challenge. Evidence suggests that psychological distress, more than medical prognosis or financial constraints, is a key factor leading patients to withdraw prematurely [29,30]. Patients sometimes terminate therapy not only because of an unfavorable medical prognosis, but also due to the accumulated emotional toll from recurrent cycles, uncertainty, and unfulfilled aspirations. This highlights a significant deficiency in traditional fertility services: the insufficient integration of organized mental and physical support into standard care. The absence of comprehensive approaches may negatively affect treatment adherence, patient engagement, and long-term well-being, emphasizing the need for more inclusive, patient-centered infertility care models [31,32].

In this study, we consolidate existing evidence to propose a more holistic paradigm for infertility therapy that integrates rehabilitation with ART. We focus on three interconnected areas: the psychological impact of infertility and the effectiveness of interventions such as counseling, cognitive behavioral therapy, and mindfulness-based techniques; physical rehabilitation methods, including pelvic floor therapy, structured exercise, and yoga, which address both reproductive physiology and sexual function; and integrated care pathways that combine psychological and physical rehabilitation, promoting outcomes that extend beyond conception to include mental health, relationship quality, and long-term quality of life.

## 2. Methods

This narrative review synthesizes current evidence on psychological and physical rehabilitation approaches in infertility care and their potential contributions to outcomes beyond conception. It provides a clinically relevant overview of rehabilitation strategies that address the multidimensional consequences of infertility, including psychological well-being, sexual health, physical functioning, relationship dynamics, treatment adherence, participation, and quality of life.

Relevant literature was identified through searches of PubMed, MEDLINE and Google Scholar conducted between November 2025 and January 2026, as well as by reviewing references from key publications, including clinical guidelines, consensus statements, systematic reviews, and seminal studies related to infertility and rehabilitation. Representative search terms included combinations of “infertility”, “assisted reproductive technology”, “psychological rehabilitation”, “cognitive behavioral therapy”, “mindfulness”, “couple counseling”, “sexual rehabilitation”, “pelvic floor muscle training”, “exercise therapy”, “physical activity”, “male infertility”, “polycystic ovary syndrome”, “yoga”, “digital health”, “quality of life”, “rehabilitation”, and “functioning”.

Publications were selected for their relevance to infertility-related functioning, psychosocial well-being, sexual health, physical rehabilitation, and integrated models of care. Greater emphasis was given to clinical guidelines, consensus statements, systematic reviews, meta-analyses, randomized controlled trials, and large observational studies. Foundational studies were included when important for understanding the evolution of infertility care, rehabilitation principles, or psychosocial outcomes.

Evidence was synthesized narratively and organized into thematic domains reflecting the major rehabilitation dimensions identified in the literature, including the psychosocial burden of infertility, psychological rehabilitation interventions, physical rehabilitation strategies, sexual rehabilitation, digital interventions, and integrated models of care. Particular attention was given to outcomes related to psychological well-being, sexual function, relationship quality, treatment adherence, participation, quality of life, and reproductive outcomes.

The review was conceptually guided by the World Health Organization’s International Classification of Functioning, Disability and Health (ICF), which was used to interpret infertility not only as a reproductive disorder but also as a condition affecting body functions and structures, activities, participation, and contextual factors. This framework provided the theoretical basis for examining how rehabilitation interventions may address the broader functional and psychosocial consequences of infertility.

Although the ICF informed the conceptual interpretation of infertility-related functioning, the evidence synthesis, figures, and summary tables were organized by rehabilitation intervention domains (psychological, physical, sexual, and integrated approaches) to facilitate clinical interpretation and application. This structure was chosen to provide a practice-oriented overview of rehabilitation strategies while maintaining consistency with the broader ICF framework.

As a narrative review, this study was not conducted according to a predefined systematic review protocol. No duplicate screening process, formal risk-of-bias assessment, or quantitative evidence synthesis was performed. The objective was to provide a clinically relevant and rehabilitation-oriented synthesis of the available literature rather than an exhaustive systematic evaluation of all published evidence.

## 3. Psychosocial Burden of Infertility

Infertility is often linked to significant psychological distress, highlighting its effects beyond the biological realm. Women undergoing ART frequently experience greater mental distress, including chronic sadness, feelings of powerlessness and lack of control, and a significantly reduced quality of life compared to women in the general population [16,23,24]. The cyclical and uncertain nature of treatment often intensifies these psychological responses, increasing emotional vulnerability over time.

In addition to its emotional consequences, infertility-related stress may have biological effects through neuroendocrine and autonomic pathways. Chronic psychological stress has been associated with activation of the hypothalamic–pituitary–adrenal (HPA) axis, cortisol dysregulation, autonomic imbalance, sleep disturbances, and inflammatory responses. These changes may negatively impact reproductive physiology, sexual functioning, and treatment engagement [16,23,24]. Unsuccessful treatment cycles and ongoing reproductive difficulties may further increase psychological distress, creating a bidirectional cycle in which emotional and physiological factors interact and reinforce each other [16,23,24]. This biopsychosocial perspective supports integrated rehabilitation approaches that address both psychological well-being and physical functioning [16,23,24].

For example, in a Taiwanese survey, nearly 40% of women attending an ART clinic met diagnostic criteria for depressive or anxiety disorders [8]. Similar findings have been reported in both Western and Asian settings, indicating that the emotional burden of infertility transcends cultural boundaries, although its expression may vary according to social norms and expectations [10,13,17]. In addition to anxiety and depression, some individuals may experience significant psychological distress associated with recurrent treatment failures, pregnancy loss, invasive procedures, or distressing healthcare experiences. Recognition of these experiences and appropriate psychosocial support may therefore represent an important component of patient-centered infertility care [10,13,17].

The strain of infertility affects both the individual and the couple’s relationship. Marital dissatisfaction, loss of intimacy, and sexual strain are frequently reported, and these issues are often intensified by the cyclical nature of fertility treatment, with repeated failures increasing conflict and emotional exhaustion [28,33]. Relational difficulties are sometimes worsened by the unpredictable course of ART, where repeated treatment attempts and failures increase emotional fatigue, frustration, and interpersonal discord [16]. Over time, this accumulated stress may reduce relationship satisfaction and impair adaptive coping within the couple.

To more effectively assess the multidimensional nature of infertility-related distress, the Fertility Problem Inventory was developed as a validated psychometric instrument that evaluates stress in the personal, marital, social, and sexual domains [34]. Its use has consistently shown that infertility is not only an individual psychological burden but also a systemic stressor affecting couples, with significant variation in how partners perceive and respond to treatment-related obstacles [18]. Longitudinal research shows that couples who rely on avoidance or maladaptive coping strategies, or who have difficulty with open communication, are at significantly greater risk of persistent distress throughout their infertility journey [35,36]. These findings support the need to integrate structured psychological rehabilitation into standard fertility care, rather than treat it as optional support.

## 4. Psychological Rehabilitation Approaches

### 4.1. Cognitive Behavioral Therapy and Coping Skills Training

Cognitive behavioral therapy (CBT) is one of the most rigorously evaluated interventions for managing infertility-related distress. By targeting negative thought patterns and avoidance behaviors, CBT helps individuals reframe their experiences, develop problem-solving strategies, and adopt more adaptive coping mechanisms [37,38]. Randomized controlled trials consistently show that CBT reduces both depressive symptoms and anxiety in women undergoing infertility treatment [39,40]. These benefits are linked not only to a decrease in symptoms but also to improved emotional regulation and a greater sense of control over the infertility experience. These are important psychological factors in chronic stress situations. CBT is also associated with better overall mental health and greater adherence to treatment, indicating that it may offer behavioral and functional benefits beyond symptom relief. Group-based CBT formats are especially effective, as they provide structured skill development and foster a sense of community and peer support that counteracts the isolation many patients experience [39,41]. These results suggest that CBT is a key evidence-based component of psychological rehabilitation in infertility care. Both individual and group formats provide important and complementary clinical advantages. Beyond symptom reduction, CBT may support treatment participation, self-management, and overall functioning by enhancing coping capacity and psychological adaptation throughout the infertility journey.

In addition, evidence from meta-analyses supports these clinical observations, showing that CBT and related psychological interventions improve psychological well-being, reduce distress, and enhance coping among individuals undergoing fertility treatment [25,40]. Although some studies have reported associations between psychological interventions and pregnancy outcomes, the evidence remains inconsistent and does not support a direct causal effect. Any potential reproductive benefit is more likely mediated through improved treatment adherence, continuation, and psychological adaptation during fertility treatment [30,42]. These findings establish CBT as a crucial, evidence-based component of psychological rehabilitation in infertility treatment. It offers structured therapeutic techniques that are clinically effective and adaptable across diverse cultural, psychosocial, and healthcare settings. In addition, its combined impact on psychological adaptation and potential reproductive outcomes further underscores its importance within comprehensive fertility management frameworks.

### 4.2. Mindfulness and Acceptance-Based Approaches

Over the past decade, mindfulness-based stress reduction (MBSR) and acceptance and commitment therapy (ACT) have been increasingly adapted for patients facing infertility [43]. Unlike CBT, which focuses on cognitive restructuring, these third-wave therapies emphasize developing present-moment awareness, psychological flexibility, and acceptance of internal experiences. MBSR and ACT encourage individuals to adopt a nonjudgmental attitude toward emotions and to pursue actions that reflect personally significant values, even amid persistent uncertainty, rather than attempting to directly modify uncomfortable thoughts. Galhardo et al. demonstrated that mindfulness training enhanced fertility-related quality of life and reduced distress symptoms, underscoring the potential of these approaches to address infertility-specific stressors [27]. Consequently, MBSR and ACT are potential adjuncts within psychological rehabilitation frameworks for infertility, offering alternative and potentially synergistic pathways to enhance psychological well-being in this population [44].

Li et al. reported that mindfulness-based programs improved emotional regulation and reduced perceived stress among women undergoing IVF. Other randomized trials evaluating both online and in-person delivery support these findings [32]. The studies highlight the scalability and accessibility of mindfulness-based interventions, especially in settings where in-person psychological support is limited. Although evidence for a direct effect on pregnancy outcomes remains inconclusive, mindfulness programs consistently enhance emotional resilience, which may indirectly support persistence in fertility treatment [32,45]. The high discontinuation rates in ART, often due to psychological burden rather than medical failure, underscore the significance of this issue. By emphasizing acceptance rather than avoidance, mindfulness-based interventions help couples tolerate uncertainty and cope more effectively with the repeated cycles often inherent in ART. From a rehabilitation perspective, these improvements may enhance emotional functioning, participation in treatment, and quality of life despite ongoing uncertainty about reproductive outcomes.

### 4.3. Couple and Family-Centered Counseling

Infertility is fundamentally a shared experience, and the distress it causes often differs between partners. Women frequently report higher levels of distress, while men may underreport emotional struggles but still experience similar levels of strain. These differences may reflect a combination of biological factors, social expectations, coping patterns, and gender-related norms that influence how infertility-related distress is experienced and expressed. Although men often underreport psychological distress during infertility treatment, this is influenced by traditional masculinity norms, societal expectations of emotional control, and a tendency to suppress emotional vulnerability [13,46]. Furthermore, fertility services have historically focused more on women’s experiences, which may contribute to the under recognition of male psychological needs. Therefore, clinical care should move beyond viewing men merely as supporters of female partners and instead recognize male partners as patients with distinct psychological, sexual, and reproductive health needs that require targeted assessment and intervention [13,46]. These differences in coping styles can create tension within relationships, leading to misunderstandings and conflict [33,47].

Couple-based counseling programs aim to bridge this gap by fostering open communication, shared decision-making, and strategies for managing sexual and marital strain. Evidence shows that such dyadic interventions improve marital satisfaction, enhance intimacy, and reduce discrepancies in coping between partners [28,32,33]. Therefore, this intervention may improve psychological adjustment and treatment persistence by fostering mutual support and aligning expectations.

In addition, family- and community-based approaches are especially important in cultural contexts where childlessness is stigmatized [14]. In these contexts, the effects of infertility often extend beyond the couple, influencing family dynamics, social identity, and perceived societal roles. Social support is a well-established buffer against the negative psychological effects of infertility, reducing isolation and alleviating feelings of shame and inadequacy [32,48]. Interventions involving family members or community support networks, rather than focusing solely on the couple, can play an important role in addressing stigma and reinforcing resilience. These approaches enhance the overall effectiveness and sustainability of psychological rehabilitation in infertility care by incorporating a more comprehensive, ecological, and culturally responsive framework. These benefits extend beyond psychological well-being to support relationship functioning, communication, and participation within family and social roles.

### 4.4. Web-Based and Digital Interventions

The growing availability of digital health tools has expanded access to psychological rehabilitation. Web-based CBT platforms and online psychoeducational programs have shown significant promise in reducing distress among women undergoing IVF [32,45]. For patients in low-resource settings or those unable to access in-person psychological services, digital interventions offer an accessible and cost-effective alternative. Online platforms also provide anonymity and flexibility, which can be especially valuable for individuals who may be reluctant to seek face-to-face counseling due to stigma or time constraints [49].

**Figure 1:**
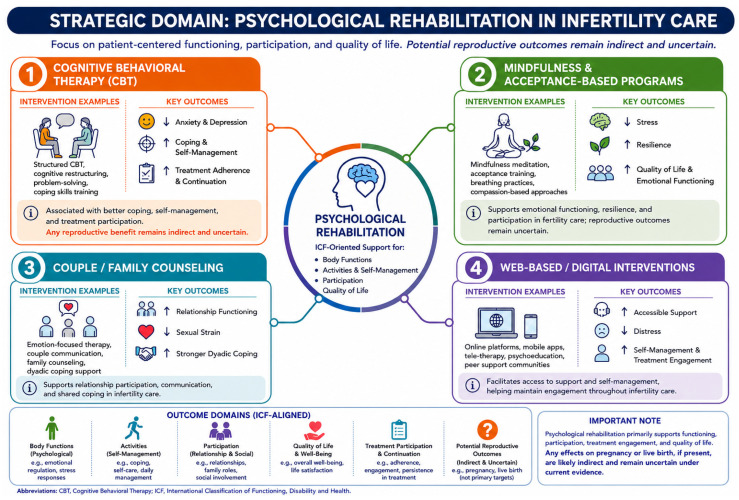
Strategic domain of psychological rehabilitation. The diagram shows four intervention pathways and their key outcomes, including cognitive behavioral therapy, mindfulness-based programs, couple/family counseling and digital interventions. ↑ indicates increase; ↓ indicates decrease.

By delivering structured, evidence-based interventions in digital formats, these tools help bridge the gap between the demand for and availability of psychological support. They also have potential for integration with other aspects of infertility rehabilitation, such as lifestyle or exercise programs, making them an important component of future models of care [42,45]. In addition to psychological support, future digital platforms may facilitate patient education, self-management, exercise guidance, and coordination of rehabilitation-related services throughout infertility treatment. However, successful implementation of digital interventions requires consideration of accessibility, health and digital literacy, language barriers, geographic disparities, and privacy protections, particularly given the sensitive nature of infertility- and sexual health-related information. Figure 1 summarizes the strategic domain of psychological rehabilitation, outlining four intervention pathways and their key outcomes. By improving access to support and self-management resources, digital interventions may facilitate ongoing engagement in treatment and participation in rehabilitation activities.

## 5. Physical Rehabilitation Approaches

### 5.1. Pelvic Floor Rehabilitation and Genito-Pelvic Pain

Pelvic floor dysfunction is an underrecognized but clinically significant contributor to infertility-related distress. Many women experiencing infertility also report sexual difficulties such as dyspareunia, vaginismus, and reduced sexual satisfaction [11]. These conditions may increase psychological stress and interfere with adherence to treatment schedules and timed intercourse. Rehabilitation strategies focused on pelvic floor health are therefore highly relevant.

Pelvic floor muscle training (PFMT), especially when combined with adjunctive modalities such as biofeedback, relaxation training, or manual therapy, has been shown to improve neuromuscular coordination, pelvic floor function, and pain modulation, as well as enhance sexual function [50,51]. These multimodal approaches address both the physiological and psychosexual components of genito-pelvic dysfunction, which are often overlooked in women undergoing infertility treatment.

Evidence from systematic reviews and meta-analyses consistently demonstrates significant improvements in Female Sexual Function Index (FSFI) scores after structured PFMT, particularly in the domains of arousal, orgasm, and satisfaction [31]. These findings indicate that PFMT improves sexual responsiveness and confidence-critical yet often overlooked components of reproductive health, and also strengthens and coordinates the pelvic floor muscles. In a randomized controlled trial, Ghaderi et al. reported that a program combining PFMT with relaxation training reduced pain severity and increased sexual satisfaction among women with dyspareunia [48]. These findings underscore that pelvic floor rehabilitation should be considered an integral component of comprehensive infertility care, particularly for women whose treatment is complicated by genito-pelvic pain disorders. These improvements may enhance sexual function, reduce activity limitations related to intercourse, and improve quality of life.

### 5.2. Exercise and Metabolic Conditioning in Women

Lifestyle modification has long been recognized as a cornerstone of infertility management, particularly in women with polycystic ovary syndrome (PCOS), one of the most common causes of anovulatory infertility [52,53]. Structured exercise interventions, including aerobic and resistance training, have consistenly been shown to improve insulin sensitivity, reduce central adiposity, and normalize reproductive endocrine function [54,55,56]. In most studies, aerobic exercise programs were performed at moderate intensity, typically corresponding to approximately 3–6 metabolic equivalents (METs), 50–70% of maximum heart rate, or a rating of perceived exertion of 11–13 on the Borg scale. Interventions commonly involved 150 min or more of weekly aerobic activity distributed across 3–5 sessions per week, consistent with widely accepted physical activity recommendations for adults. Although moderate exercise is generally beneficial, excessive endurance training and low energy availability may adversely affect reproductive function through hormonal disturbances and impaired ovulatory function. Therefore, exercise programs should be individualized and balanced to optimize health benefits while avoiding potential negative effects associated with overtraining [54,55].

Legro et al. conducted a landmark randomized trial showing that preconception lifestyle modification, including dietary changes and regular exercise, significantly improved ovulation and increased pregnancy rates in women with PCOS [55]. These findings provided some of the first high-quality evidence supporting lifestyle optimization as a treatment approach rather than merely supplementary guidance in reproductive medicine. Subsequent systematic reviews and meta-analyses have confirmed these results, highlighting benefits that extend beyond ovulation to improvements in menstrual regularity, metabolic profiles, and overall quality of life [56,57]. These multifaceted outcomes underscore the interconnected metabolic and reproductive mechanisms associated with PCOS, emphasizing the importance of comprehensive care strategies. The benefits of structured exercise are not limited to improvements in physical fitness. Exercise may contribute to reproductive health through reductions in systemic inflammation and oxidative stress, improvements in insulin sensitivity and metabolic regulation, enhancement of endothelial function, and modulation of autonomic and hormonal balance. These physiological adaptations are particularly relevant in conditions such as obesity, metabolic syndrome, and polycystic ovary syndrome, where metabolic and reproductive dysfunction are closely interconnected [56,57]. Based on this evidence, recent Cochrane reviews recommend lifestyle modification, particularly exercise, as a first-line approach for overweight and obese women with PCOS who wish to conceive [57]. These findings position exercise and metabolic conditioning as more than adjunctive strategies; they are evidence-based rehabilitation interventions that directly influence reproductive physiology, enhance the effectiveness of fertility treatments, and support long-term health outcomes in women. In addition to reproductive outcomes, exercise interventions may improve physical functioning, daily activity performance, and health-related quality of life.

### 5.3. Exercise and Reproductive Health in Men

The role of physical activity in male reproductive health has received increasing attention in recent years. Sedentary behavior, obesity, and metabolic syndrome are strongly associated with impaired semen quality, hormonal imbalance, and elevated oxidative stress, all of which contribute to male infertility [12,58]. Rehabilitation strategies emphasizing moderate aerobic exercise have been shown to improve sperm concentration, motility, and morphology, as well as enhance testosterone levels and reduce markers of oxidative stress [58,59,60].

Sharma et al. highlighted that lifestyle modification, including exercise, nutrition, and weight management, can significantly improve male reproductive parameters and should be considered a primary rehabilitation strategy for men undergoing infertility evaluation [58]. However, it is important to recognize that excessive endurance training or extreme exercise loads may negatively affect sperm quality and testosterone production. These findings suggest the need for individualized rehabilitation programs that promote balanced, sustainable exercise regimens tailored to the reproductive goals of male patients. This approach ensures that lifestyle therapies enhance reproductive outcomes while preventing unexpected negative effects, reinforcing their importance in the holistic management of male infertility. These benefits may support broader physical functioning, health maintenance, and participation in fertility treatments.

### 5.4. Yoga and Mind–Body Approaches

Mind–body interventions, particularly yoga, have become increasingly integrated into infertility rehabilitation programs because of their combined psychological and physiological benefits. Yoga incorporates controlled breathing, mindfulness, and structured physical postures, which together reduce perceived stress, regulate autonomic balance, and improve overall well-being [61]. Randomized controlled trials have shown that yoga can significantly reduce anxiety, perceived stress, and psychological distress among women undergoing ART [46,62]. These benefits may improve emotional resilience and support continued engagement with fertility treatment. However, current evidence is insufficient to conclude that yoga directly improves pregnancy outcomes, and any reproductive benefit should be considered an indirect possibility rather than a primary therapeutic effect.

For example, it has been reported that women who participated in yoga interventions during IVF cycles had lower stress scores and greater psychological resilience compared with control groups [62]. These findings indicate that yoga may be an effective supplementary approach to alleviate mental distress associated with reproductive treatment, especially during periods of uncertainty and repeated procedures.

Beyond psychological benefits, yoga may influence physiological stress responses through mechanisms such as autonomic regulation and cortisol modulation [63]. However, the clinical significance of these mechanisms for reproductive outcomes remains uncertain. The findings indicate that yoga may be an effective supplementary approach to alleviate mental distress associated with reproductive treatment, especially during periods of uncertainty and repeated procedures [64]. Given its accessibility, adaptability across cultures, and relatively low cost, yoga is a practical and widely applicable rehabilitation strategy for couples undergoing infertility treatment. Consequently, yoga may support emotional functioning, treatment participation, and overall well-being in individuals receiving infertility care.

### 5.5. Sexual Rehabilitation for Couples

Sexual dysfunction is common among couples facing infertility and may manifest as reduced libido, erectile dysfunction, difficulties with arousal, or lack of intimacy. These issues are not merely secondary concerns but can actively undermine treatment adherence, marital stability, and overall quality of life. Rehabilitation programs that address sexual health are therefore highly important.

Evidence supports the use of structured sexual counseling, sensate focus exercises, and CBT-based sexual therapy to improve both marital satisfaction and sexual functioning [65]. This approach addresses not only sexual performance issues but also maladaptive thoughts, performance anxiety, and avoidance behaviors, which often lead to sexual distress in this population. A randomized controlled trial demonstrated that CBT-based sexual counseling improved sexual satisfaction and strengthened marital relationships among infertile women [33].

For men, interventions such as pelvic floor physiotherapy, behavioral therapies, and targeted counseling have been effective in managing erectile dysfunction and premature ejaculation, which are often sources of stress and reduced treatment success [58,66,67]. These conditions often create a cycle of performance anxiety and reduced sexual confidence, increasing relational tension. Male patients may also have rehabilitation needs that go beyond sexual performance alone. Semen collection procedures, concerns about masculinity and fertility, stigma related to male-factor infertility, and performance anxiety associated with timed intercourse or assisted reproduction can cause distress, reduce treatment engagement, and impair quality of life. Therefore, counseling and sexual rehabilitation programs should recognize male partners as patients with distinct psychosocial and functional needs, not just as supporters of female infertility treatment.

Furthermore, Flynn et al. emphasized that sexual health is a critical determinant of well-being throughout the life course, making it an essential dimension of infertility rehabilitation rather than a peripheral issue [68]. This perspective underscores the importance of including sexual rehabilitation in standard treatment protocols, making it a crucial component of holistic, couple-centered infertility management rather than a secondary concern. Rehabilitation of sexual function may reduce participation restrictions that affect intimacy, partner relationships, and quality of life.

### 5.6. Preventive Role of Pelvic Floor Muscle Training

**Figure 2:**
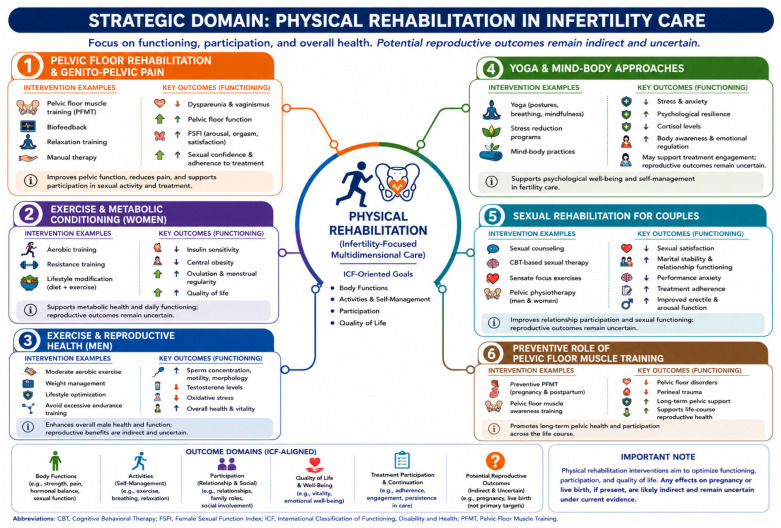
Strategic domain of physical rehabilitation in infertility care. The figure summarizes physical rehabilitation interventions that support functioning, symptom management, sexual health, physical capacity, and treatment participation among individuals and couples experiencing infertility. Core infertility-focused interventions include pelvic floor rehabilitation, exercise-based rehabilitation, sexual rehabilitation, and mind–body approaches. Preventive pelvic floor muscle training is presented as a broader life-course pelvic health strategy that may support long-term reproductive well-being but is not a direct infertility treatment. Reproductive outcomes remain indirect and uncertain. ↑ indicates increase; ↓ indicates decrease.

While pelvic floor training is often introduced as a therapeutic measure for existing dysfunction, its preventive value also deserves recognition. Evidence indicates that structured PFMT during pregnancy and postpartum reduces the risk of pelvic floor disorders, minimizes perineal trauma, and improves long-term pelvic support [31,69]. These benefits result from improvements in neuromuscular control, muscle strength, and connective tissue support, all of which help maintain pelvic stability. Although not directly related to infertility treatment, these findings highlight the broader role of pelvic rehabilitation in protecting reproductive health throughout the female life course. By integrating preventive strategies, clinicians can reduce the likelihood of later complications, supporting reproductive wellness in both immediate and long-term contexts. This approach enables a shift from a purely reactive care model to a more preventive, integrative, and life-course-oriented framework for pelvic rehabilitation. This preventive approach aligns with rehabilitation goals of maintaining function and minimizing future disability throughout the reproductive life course. Figure 2 illustrates the strategic domain of physical rehabilitation, including core physical interventions and their corresponding outcomes. Outcomes shown in the figure are organized as: (1) primary physical functioning outcomes, (2) secondary psychosocial outcomes, and (3) indirect reproductive outcomes where evidence remains uncertain.

## 6. Rationale for Integration Approach

Infertility cannot be meaningfully divided into medical, psychological, or physical components. Instead, it should be understood as a complex condition involving the interaction of reproductive physiology, psychological well-being, physical functioning, sexual health, lifestyle factors, and social participation [42,68,70]. Based on principles of rehabilitation and the International Classification of Functioning, Disability and Health (ICF) [42,68,70], there is an Integrated Infertility Rehabilitation Model, which includes psychological, physical, sexual, and lifestyle rehabilitation as essential elements of comprehensive infertility care. This model may enhance functioning, resilience, relationship quality, and overall quality of life. In addition to psychosocial consequences, growing evidence suggests that infertility-related distress may influence reproductive outcomes through interconnected biological pathways.

Chronic psychological stress has been linked to dysregulation of the HPA axis, changes in cortisol secretion, autonomic imbalance, sleep and circadian disturbances, and increased systemic inflammatory and oxidative stress responses [42,68,70]. Such mechanisms may adversely affect reproductive physiology, including ovulatory function, endometrial receptivity, sexual function, spermatogenesis, and treatment adherence. Conversely, interventions targeting psychological well-being, physical activity, sleep quality, metabolic health, and sexual functioning may exert complementary effects on both psychosocial adaptation and reproductive health. This bidirectional interaction provides a biological rationale for an integrated infertility rehabilitation model in which psychological, physical, sexual, and lifestyle interventions are delivered in parallel with conventional reproductive medicine [28,32,33,48,71].

This interplay creates a cycle in which psychological strain worsens physical symptoms, while physical dysfunction increases distress. Integrated rehabilitation seeks to break this cycle by addressing both domains simultaneously. For example, cognitive behavioral therapy or mindfulness programs can reduce anxiety and improve motivation, increasing adherence to structured exercise or pelvic floor interventions [27,32,35]. Conversely, improvements in sexual functioning and physical well-being often restore self-confidence, elevate mood, and enhance couple dynamics [13,31,72]. These bidirectional benefits provide a strong rationale for designing rehabilitation models that address infertility holistically rather than in a piecemeal manner.

### 6.1. Models of Integrated Care

Several practical approaches demonstrate how rehabilitation can be systematically integrated into fertility services. Mind–body programs developed by Domar and colleagues used structured group interventions that combined relaxation training, cognitive restructuring, stress management skills, and lifestyle counseling. Their results showed reductions in psychological distress and higher reported pregnancy rates among women undergoing IVF in some settings [26,39]. However, the most consistent benefits of integrated mind–body interventions are improvements in psychological well-being, coping, and treatment engagement. Any observed reproductive benefits should be interpreted cautiously, as they may be mediated through improved treatment adherence and reduced treatment discontinuation rather than a direct biological effect of the intervention. Routine psychosocial care guidelines from professional societies such as the European Society of Human Reproduction and Embryology formally recommend including psychosocial care at all stages of medically assisted reproduction. These guidelines emphasize that psychological and physical rehabilitation should be routine components delivered by trained fertility staff alongside biomedical interventions, not optional extras [73]. Implementation of integrated infertility rehabilitation will likely require collaboration among reproductive specialists, mental health professionals, rehabilitation practitioners, and other allied health professionals, with the specific composition of the team varying according to local resources, patient needs, and healthcare system structures [73,74].

Lifestyle and rehabilitation interventions for women with PCOS, including comprehensive programs that combine dietary counseling, structured exercise, and behavioral strategies have shown improvements in ovulation and increased pregnancy rates [54,55,56,57]. These integrated protocols demonstrate that physical rehabilitation can be effectively combined with psychological support to improve both health behaviors and reproductive outcomes. Several of these interventions also align with broader preconception care principles, including lifestyle optimization, management of chronic health conditions, mental health support, paternal health promotion, and patient education. Although infertility rehabilitation and preconception care are distinct frameworks, they share the common goal of improving health and well-being before and during fertility treatment [54,55,56,57]. Couple-based approaches, such as couple counseling, sexual rehabilitation, or dyadic coping strategies, can reduce marital strain and strengthen intimacy. These relational improvements are important, as a supportive partner dynamic can indirectly influence treatment persistence and overall success [13,32,33]. Digital health innovations further enhance integration. Web-based CBT platforms can be linked with tele-physiotherapy or online lifestyle coaching, creating blended programs that are scalable and accessible across geographic or economic barriers [42,45]. These hybrid models may be especially valuable in resource-limited settings, where access to in-person multidisciplinary care is limited.

**Figure 3:**
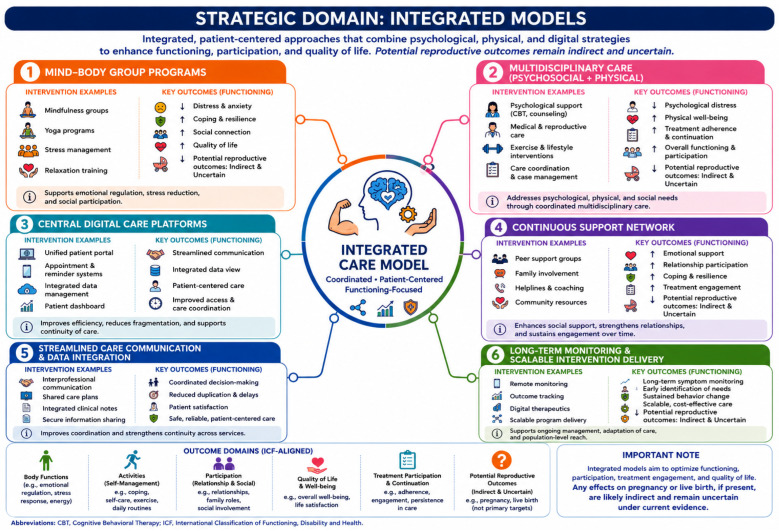
Models for integrated care. This diagram summarizes the synergy between psychosocial and physical care. It illustrates how multidisciplinary care, mind–body programs, and digital platforms combine to reduce distress, improve pregnancy rates, and provide holistic support beyond conception. ↑ indicates increase; ↓ indicates decrease.

In addition, sleep and circadian health may be important components of comprehensive infertility rehabilitation. Emerging evidence suggests that sleep disturbances and circadian rhythm disruption may influence stress regulation, endocrine function, metabolic homeostasis, and reproductive hormone balance. Although evidence on specific rehabilitation interventions is limited, assessing sleep health may complement broader psychological, lifestyle, and physical rehabilitation strategies within integrated infertility care models [42,45]. Figure 3 shows models for integrated care, summarizing the synergy between psychosocial and physical care.

### 6.2. Outcomes Beyond Conception

Traditionally, the success of infertility treatment has been measured almost exclusively by pregnancy and live birth rates. While these outcomes are undeniably important, this narrow focus overlooks the broader impact of infertility and the various ways rehabilitation contributes to patient well-being. For many couples, the treatment journey—often marked by repeated cycles, emotional highs and lows, and financial strain—can leave lasting psychological and relational consequences, regardless of whether pregnancy is achieved.

Rehabilitation broadens the scope of outcome evaluation. Improvements in mental health, reduced anxiety and depression, restored sexual intimacy, and stronger couple relationships are outcomes that hold significant value for patients, regardless of conception. Patient-centered studies consistently show that individuals who receive psychosocial support report higher satisfaction with care and lower treatment-related distress, even when fertility outcomes are unfavorable [56,74]. Furthermore, social support and positive dyadic coping strategies predict more adaptive long-term adjustment, indicating that rehabilitation provides benefits that extend well beyond the immediate treatment period [35,45].

Additional rehabilitation-focused outcomes should be considered beyond pregnancy and live birth rates. These include treatment adherence, psychological resilience, sexual well-being, relationship satisfaction, self-efficacy, and long-term mental health. Such outcomes may affect patients’ ability to cope with prolonged treatment journeys and may remain clinically meaningful regardless of reproductive outcome [35,45].

In this context, integrated rehabilitation redefines success in infertility care. Rather than focusing solely on biological parenthood, it helps couples maintain resilience, preserve their relationships, and protect their quality of life [19]. Whether patients conceive through ART, choose adoption, or remain child-free, rehabilitation ensures they emerge from the infertility experience with greater psychological strength and relational stability.

## 7. Synthesis of Findings

Taken together, the three strands of evidence addressed in this review converge on several key insights. First, psychological rehabilitation through CBT, mindfulness, and couple-centered interventions consistently reduces anxiety and depression, improves coping, enhances quality of life, strengthens relationship functioning, and supports treatment adherence and continuation [25,39,40,41,42,45]. Although some studies have reported associations with improved pregnancy outcomes, the evidence remains inconsistent, and any reproductive benefit is likely indirect rather than a direct effect of the interventions. Second, physical rehabilitation—including pelvic floor training, structured exercise for women with PCOS, moderate activity for men, and mind–body practices such as yoga—can directly support reproductive physiology and alleviate sexual dysfunction while also contributing to quality of life [12,31,48,54,55,56,57,58]. Third, integrated care models that combine psychological and physical approaches demonstrate the strongest potential, as they address the bidirectional interaction among stress, physical dysfunction, and reproductive success [13,26,27,32,33,42,54,55,56,57,73]. Table 1 provides an overview of these findings, summarizing the main rehabilitation domains, associated interventions, expected outcomes, and representative sources. This synthesis highlights how psychological, physical, and integrated models complement each other, reinforcing the need for a holistic approach to infertility care.

## 8. Study Limitations

Several limitations of this review should be acknowledged. First, this study was conducted as a narrative review rather than a systematic review or meta-analysis. Although the literature search was designed to identify clinically relevant evidence across multiple rehabilitation domains, formal systematic review procedures such as duplicate screening, predefined eligibility criteria, protocol registration, and quantitative risk-of-bias assessment were not performed. Therefore, the findings should be interpreted as a narrative synthesis of the available evidence rather than a comprehensive evaluation of all published studies.

Second, the evidence base for infertility rehabilitation is diverse. Psychological interventions, such as cognitive behavioral therapy and mindfulness-based approaches, are supported by multiple systematic reviews and meta-analyses. In contrast, evidence for various physical and integrated rehabilitation strategies comes from a combination of randomized controlled trials, observational studies, and narrative literature. Differences in study populations, intervention methods, outcome measures, and reproductive treatment settings may limit direct comparisons between studies.

Third, although many rehabilitation therapies have consistently demonstrated benefits for psychological well-being, sexual function, treatment adherence, and quality of life, the evidence for direct associations with improved pregnancy or live birth outcomes is less consistent. Improvements in reproductive outcomes may occur indirectly through reduced psychological distress, better coping, healthier lifestyle practices, and increased treatment adherence. Therefore, reproductive benefits should be interpreted with appropriate caution.

Finally, infertility is influenced by various biological, psychological, social, cultural, and health system factors. Although this evaluation was guided by the ICF, the use of rehabilitation treatments may vary by geographic region, healthcare system, resource environment, and cultural context. Future research should focus on developing and evaluating standardized multidisciplinary rehabilitation pathways and their effects on patient-centered and reproductive outcomes. Research should also investigate the implementation of rehabilitation-oriented reproductive care across the life course, including adolescents and young adults at risk for future infertility, those requiring fertility preservation, and populations with reproductive conditions that could affect long-term fertility and reproductive health.

An additional limitation of this review is that many included studies did not consistently distinguish between biological sex and gender-related factors or report sex-specific outcomes in sufficient detail. Consequently, the ability to evaluate potential differences in rehabilitation needs, experiences, and responses between men and women remains limited. Future research should include more explicit reporting of sex- and gender-related variables to improve understanding of their influence on infertility rehabilitation outcomes.

## 9. Conclusions

Infertility is more than a medical diagnosis; it is a multidimensional challenge that affects emotional health, physical functioning, and relationships. Although advances in assisted reproductive technologies have expanded treatment options, many individuals discontinue care because of psychological strain, relationship difficulties, or reduced quality of life. Rehabilitation is an essential complement to medical treatment. Psychological approaches such as counseling, cognitive behavioral therapy, and mindfulness strengthen resilience, reduce distress, improve quality of life, and support continued engagement with fertility treatment, while physical strategies including pelvic floor training, structured exercise, yoga, and sexual rehabilitation enhance well-being, sexual functioning, and intimacy. Together, these interventions create a holistic model of care that supports adherence, restores confidence, and redefines success beyond conception. A patient-centered approach to infertility treatment should therefore measure outcomes not only by pregnancy rates but also by improved mental health, stronger partnerships, and sustained quality of life. By integrating rehabilitation into fertility care, clinicians can help patients navigate their journey with greater strength, dignity, and balance.

## Figures and Tables

**Table 1:** Summary of Rehabilitation Approaches in Infertility Care.

Domain	Intervention	Target Population	Key Outcomes	Evidence Type	Certainty of Evidence	References
Psychological Rehabilitation	Cognitive Behavioral Therapy (CBT)	General infertility patients; women undergoing infertility treatment or ART	↓ Anxiety & depression; ↑ coping; modest ↑ in pregnancy rates	Systematic reviews, meta-analyses, randomized controlled trials	Strong	[25,39,40,41]
	Mindfulness & Acceptance-Based Programs	Infertility patients experiencing psychological distress; women undergoing IVF/ART	↓ Stress; ↑ resilience; better quality of life	Randomized controlled trials, systematic reviews	Moderate	[27,32,45]
	Couple/Family Counseling	Couples experiencing infertility-related relational distress, communication difficulties, or marital strain	↑ Marital satisfaction; ↓ sexual strain; stronger dyadic coping	Clinical studies, observational studies, randomized trials	Limited	[28,32,33,48]
	Web-Based/Digital Interventions	Infertility patients with limited access to in-person services or requiring flexible support	Accessible support; ↓ distress; scalable delivery	Randomized trials, observational studies, systematic reviews	Moderate	[42,45]
Physical Rehabilitation	Pelvic Floor Muscle Training (PFMT)	Women with dyspareunia, vaginismus, pelvic floor dysfunction, or infertility-associated sexual pain	↑ Sexual function (FSFI); ↓ dyspareunia	Systematic reviews, meta-analyses, randomized controlled trials	Strong	[31,48]
	Exercise in Women with PCOS	Women with PCOS, obesity, overweight, or metabolic dysfunction associated with infertility	↑ Ovulation; ↑ pregnancy; improved metabolic profile	Randomized controlled trials, systematic reviews, meta-analyses	Strong	[54,55,56,57]
	Exercise in Men	Men with obesity, sedentary lifestyles, metabolic syndrome, or impaired semen parameters	↑ Sperm quality; ↑ testosterone; ↓ oxidative stress	Clinical studies, observational studies, systematic reviews	Limited	[12,58]
	Yoga & Mind–Body Practices	Women undergoing ART/IVF and infertility patients experiencing stress or psychological burden	↓ Stress & anxiety; ↑ resilience; improved psychological well-being; reproductive benefits remain uncertain	Randomized controlled trials, systematic reviews	Limited	[62,63]
	Sexual Rehabilitation	Couples experiencing sexual dysfunction, reduced intimacy, erectile dysfunction, premature ejaculation, or sexual distress	↑ Sexual satisfaction; ↑ intimacy; ↓ dysfunction	Clinical studies, randomized trials, observational studies	Moderate	[33,58,68]
Integrated Models	Mind–Body Group Programs	Infertility patients experiencing psychological distress	↓ Distress; ↑ pregnancy rates	Clinical intervention studies	Moderate	[26,39]
	Multidisciplinary Care (psychosocial + physical)	General infertility patients with multidimensional rehabilitation needs	Holistic outcomes beyond conception	Guideline and review evidence	Moderate	[13,54,55,56,57,73]
	Digital + Hybrid Programs	Infertility patients requiring blended rehabilitation support	Blended CBT, lifestyle, and physiotherapy support	RCT + Systematic review and meta-analysis	Moderate	[42,45]

↑ indicates increase; ↓ indicates decrease.

## Data Availability

No new data were created or analyzed in this study. Data sharing is not applicable to this article.

## Abbreviations

The following abbreviations are used in this manuscript:

ART	Assisted reproductive technologies
IVF	In vitro fertilization
ICSI	Intracytoplasmic sperm injection
CBT	Cognitive behavioral therapy
MBSR	Mindfulness-based stress reduction
ACT	Acceptance and commitment therapy
PFMT	Pelvic floor muscle training
PCOS	Polycystic ovary syndrome
ICF	International Classification of Functioning, Disability and Health
HPA	Hypothalamic–pituitary–adrenal
